# Profiling of Early Immune Responses to Vaccination Using THP-1-Derived Dendritic Cells

**DOI:** 10.3390/ijms25105509

**Published:** 2024-05-18

**Authors:** Lei Ye, Ping Li, Mingzhe Wang, Feng Wu, Sanyang Han, Lan Ma

**Affiliations:** 1 Institute of Biopharmaceutical and Health Engineering, Tsinghua Shenzhen International Graduate School, Tsinghua University, Shenzhen 518055, China; yelei@szbl.ac.cn (L.Y.); lipinga104@163.com (P.L.); wang-mz17@mails.tsinghua.edu.cn (M.W.); wf19@mails.tsinghua.edu.cn (F.W.); 2 Institute of Biomedical Health Technology and Engineering, Shenzhen Bay Laboratory, Shenzhen 518052, China; 3 State Key Laboratory of Chemical Oncogenomics, Tsinghua Shenzhen International Graduate School, Tsinghua University, Shenzhen 518055, China

**Keywords:** immunogenicity, biomarker, systems vaccinology, transcriptomics, SARS-CoV-2

## Abstract

The COVID-19 pandemic has made assessing vaccine efficacy more challenging. Besides neutralizing antibody assays, systems vaccinology studies use omics technology to reveal immune response mechanisms and identify gene signatures in human peripheral blood mononuclear cells (PBMCs). However, due to their low proportion in PBMCs, profiling the immune response signatures of dendritic cells (DCs) is difficult. Here, we develop a predictive model for evaluating early immune responses in dendritic cells. We establish a THP-1-derived dendritic cell (TDDC) model and stimulate their maturation in vitro with an optimal dose of attenuated yellow fever 17D (YF-17D). Transcriptomic analysis reveals that type I interferon (IFN-I)-induced immunity plays a key role in dendritic cells. IFN-I regulatory biomarkers (IRF7, SIGLEC1) and IFN-I-inducible biomarkers (IFI27, IFI44, IFIT1, IFIT3, ISG15, MX1, OAS2, OAS3) are identified and validated in vitro and in vivo. Furthermore, we apply this TDDC approach to various types of vaccines, providing novel insights into their early immune response signatures and their heterogeneity in vaccine recipients. Our findings suggest that a standardizable TDDC model is a promising predictive approach to assessing early immunity in DCs. Further research into vaccine efficacy assessment approaches on various types of immune cells could lead to a systemic regimen for vaccine development in the future.

## 1. Introduction

The COVID-19 pandemic caused by SARS-CoV-2 has highlighted the critical importance of rapid vaccine development and deployment to control infectious diseases. The typical vaccine development timeline can take 10–15 years from initial discovery to commercial launch [1]. However, in the case of SARS-CoV-2 vaccines, this process, which encompassed humoral and cellular immune response studies [2,3] and clinical trials [4,5], was compressed into less than one year [6]. This compressed timeline has also raised questions about the long-term safety and efficacy of these vaccines. SARS-CoV-2 elicits a weak adoptive immune response in patients [7], and the total agreement between the neutralizing activity of anti-SARS-CoV-2 serum and the serum total IgG of patients is weak [8]. In this context, it is important to establish rapid and effective methods for vaccine assessment. In vitro neutralizing antibody levels can predict protection against symptomatic infection of SARS-CoV-2, but neutralizing antibody titers have typically been performed several weeks after the first dose of vaccination [9]. On the other hand, innate immunity plays a fundamental role in the early stage of infection and vaccination, and its associated biomarkers can predict the immune response in the short term [10]. Therefore, a valid assessment of vaccine performance using innate-immunity-associated biomarkers can be complementary to neutralizing antibody testing [11].

In the past decade, there has been promising progress in profiling immune responses [12] and assisting in vaccine design [13] using systems vaccinology approaches. Notably, systems biological assessments profile the immune signatures of pathogen infection [14] and vaccination [15] to identify biomarkers assessing immune responses. These approaches focus on human PBMCs containing various immunocytes [16]. The omics data obtained from PBMCs present the post-average signatures of several types of immunocytes, and the differentially expressed genes of less numerous but functionally important cells, such as DCs, are possibility omitted. DCs are antigen-presenting cells (APCs) that play important roles in the initiation of primary immune responses, the induction of immunological tolerance, and mediation of adaptive immunity [17,18]. The classification of DCs includes classical type 1 and type 2 DCs, plasmacytoid DCs, Langerhans cells, pre-DCs, monocyte-derived DCs (moDCs), and non-classical monocytes [19]. MoDCs can be induced from monocytes [20,21], stimulated into mature DCs (mDCs) [22], and function in the innate sensing of pathogens [23]. Lately, moDCs have received increasing attention because they play a critical role in the immune response by serving as key antigen-presenting cells that bridge innate and adaptive immunity, stimulating T cell activation and differentiation and contributing to immune regulation, inflammation, and potential therapeutic interventions [24]. Another advantage of DC biomarkers is that the innate immune response usually peaks within a week [25], unlike biomarkers of acquired immunity such as B cells and T cells, which take more than a month to be significantly upregulated [26]. However, systemic vaccinology studies have reported few DC biomarkers for vaccine assessment, mainly because (1) DCs are underrepresented in PBMCs, and the differences in the gene expression of DCs identified in PBMC samples using conventional RNA sequencing would be unremarkable; and (2) standardized vaccine evaluation using human moDCs is difficult due to the heterogeneity of the immune system [27].

In this study, we established a standardizable approach to early immune response assessment of vaccines using a THP-1-derived dendritic cell (TDDC) model. A live-attenuated yellow fever vaccine YF-17D was used to stimulate the TDDCs. Transcriptomic analysis of the TDDCs stimulated with the optimal dose of YF-17D revealed that the IFN-I-induced immunity was predominant. Ten gene signatures describing this mechanism were identified and validated. The expressions of these biomarkers in blood samples from YF-17D-vaccinated mice and humans [28] were consistent with the results from the TDDCs, demonstrating their potential for clinical application. Moreover, we applicated this assessment approach to PBMC samples from BBIBP-CorV inactive SARS-CoV-2 vaccine recipients and datasets from systems vaccinology studies on other vaccines to reveal the relevant immune response profile elicited by different types of vaccines. Collectively, we established a TDDC model for early immune response assessment in DCs, identified ten biomarkers of innate immunity, and explored the potential of their application in clinical blood testing, hoping to provide novel insights into the profiling of early immune responses in vaccine assessment.

## 2. Results and Discussion

### 2.1. Stimulation of the TDDCs Using the Optimal Dose of YF-17D In Vitro

**Figure 1:**
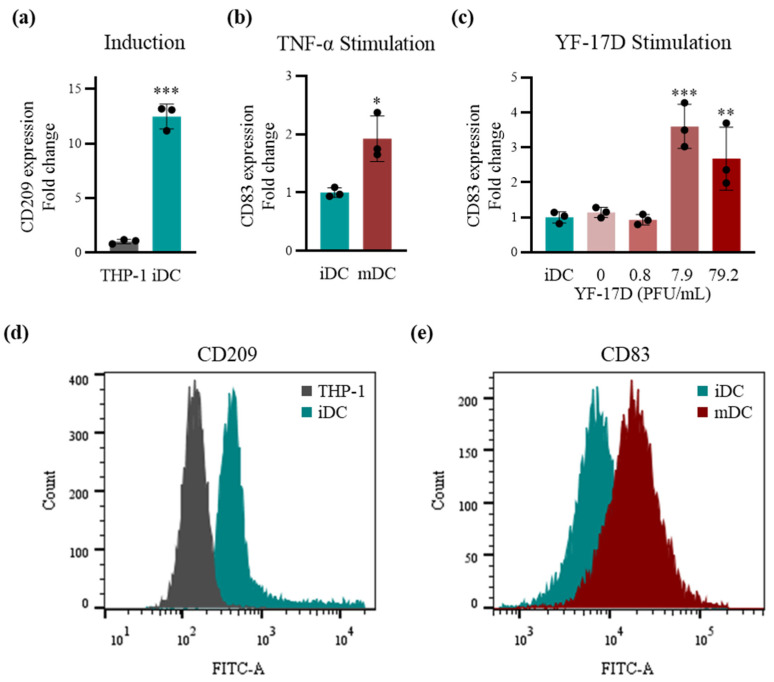
Establishment of a THP-1-derived dendritic cell (TDDC) model. (**a**) Relative expression of CD209 mRNA in THP-1 cells and THP-1-derived immature dendritic cells (iDCs) via qRT-PCR (*n* = 3). (**b**) Relative expression of CD83 mRNA in THP-1-derived iDCs and TNF-α-stimulated mature dendritic cells (mDCs) via qRT-PCR (*n* = 3). (**c**) Relative expression of CD83 mRNA in THP-1-derived iDCs and dendritic cells stimulated by PBS and 0.8, 7.9, and 79.2 PFU/mL of YF-17D via qRT-PCR (*n* = 3). (**d**) Relative expression of CD209 protein in THP-1 cells and THP-1-derived iDCs via flow cytometry. (**e**) Relative expression of CD83 protein in THP-1-derived iDCs and dendritic cells stimulated by 7.9 PFU/mL of YF-17D via flow cytometry. Data are represented as means ± SD. Statistical significance in (**a**) and (**b**) was determined using a two-tailed Student’s *t*-test. Statistical significance in (**c**) was calculated via one-way analysis of variance (ANOVA) with Dunnett’s test. * *p* < 0.05, ** *p* < 0.01, and *** *p* < 0.001.

The THP-1 cell line has previously been induced into immature DCs (iDCs) [29] for the investigation of immune responses [30,31,32]. After optimization, we determined the induction protocol of THP-1 using 100 ng/mL of IL-4 and 100 ng/mL of GM-CSF, starting with a cell density of 500 thousand per mL, exchanging the medium every two days with fresh cytokine-supplemented medium, and maintaining a cell count of less than 2 million per mL. To ensure successful modeling of the TDDCs (Figure 1), we chose CD209 as the biomarker for THP-1 induction and CD83 for iDC maturation. CD209 is a DC-specific C-type lectin [22] that is barely expressed in monocytes but abundantly expressed in DCs. CD209 was significantly increased in the THP-1-derived iDCs compared to THP-1 at both the transcriptomic (Figure 1a) and protein levels (Figure 1d and Figure S1), demonstrating successful induction. The TD-iDCs were harvested and resuspended in serum-free medium with 200 ng/mL of rhIL-4, 100 ng/mL of rhGM-CSF, 20 ng/mL of rhTNF-α, and 200 ng/mL of ionomycin and continued to be stimulated for 48 h into THP-1-derived mDCs. CD83 inhibits T cell proliferation after T cell maturation [33], and therefore CD83+ DCs stimulated from monocytes present the morphological and functional characteristics of mDCs [34]. Quantitative detection showed that the expression of CD83 was statistically increased (Figure 1b), suggesting that the THP-1-derived iDCs had a tendency to mature in response to stimulation.

Next, a classical vaccine was selected for optimizing the stimulation dose of the TDDCs. The YF-17D yellow fever vaccine has been administered to hundreds of millions of people and proven safe and effective [35]. Moreover, the immune responses to this vaccine have been thoroughly studied using systems vaccinology approaches on human PBMCs [28,36]. Therefore, we added YF-17D at concentrations of 0.8, 7.9, and 79.2 PFU/mL to the culture medium to stimulate the aforementioned THP-1-derived iDCs. The relative expression of CD83 showed that stimulation at 7.9 PFU/mL led to the peak at the transcriptional level (Figure 1c), and maturation was also confirmed at the protein level (Figure 1e and Figure S2). Meanwhile, we examined the expression of CD86 to corroborate the maturation of the DCs via qPCR (Figure S3). The results showed that the expression of CD86 was significantly upregulated in comparison to that in the iDCs under the stimulation of YF-17D at concentrations of 7.9, 79.2, and 792.4 PFU/mL, but the differences in expression between various YF-17D doses were not significant. At this point, we had successfully established a TDDC model and optimized the vaccine dose to stimulate the maturation of iDCs into mDCs in vitro.

### 2.2. Predominance of IFN-I-induced Immunity in Early Immune Responses of TDDCs

**Figure 2:**
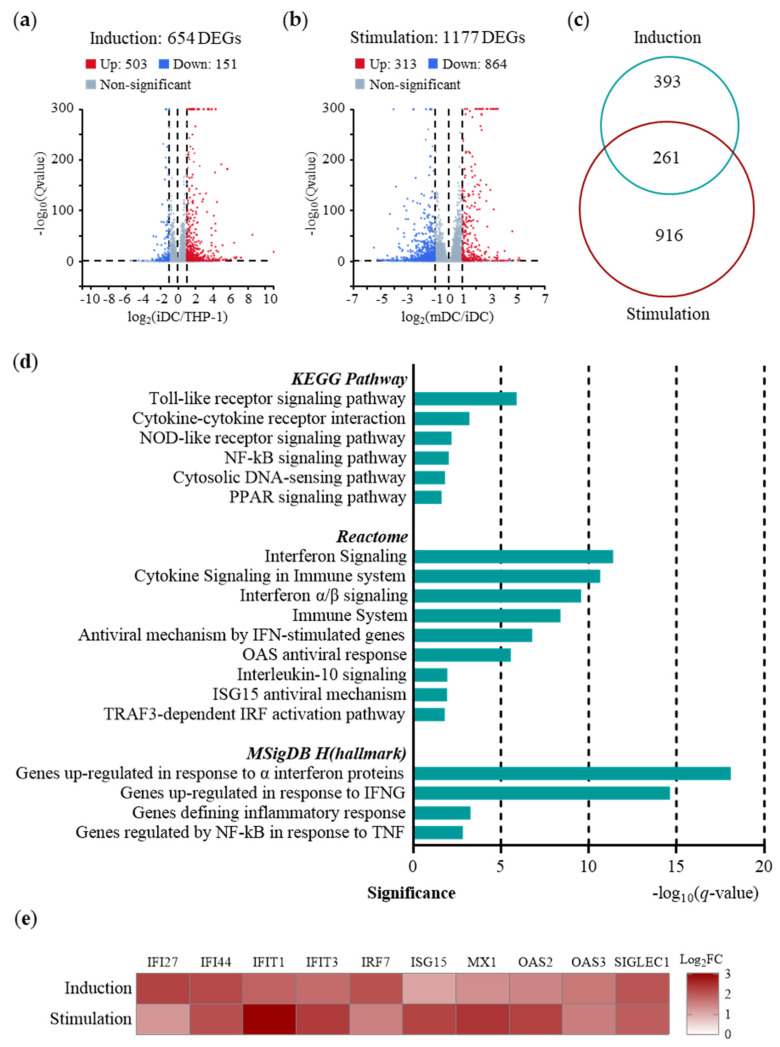
Transcriptomic profiling of TDDCs stimulated by YF-17D in vitro (*n* = 3). (**a**) Volcano map of differently expressed genes (DEGs) in induction and (**b**) YF-17D stimulation. DEGs were identified using Log2FC > 1 or < −1 and *q*-value < 0.05. Upregulated DEGs are shown in red, while downregulated DEGs are shown in blue. (**c**) Venn map of DEGs identified in induction and YF-17D stimulation. (**d**) Kyoto Encyclopedia of Genes and Genomes (KEGG), Reactome, and Molecular Signatures Database Hallmark Gene Sets (MSigDB_H) analyses of upregulated DEGs in YF-17D stimulation. (**e**) Heatmap of gene signatures from essential pathways in YF-17D-stimulated TDDCs. Relative expressions were obtained via bulk RNA sequencing.

Systems vaccinology studies have revealed that the innate immune responses to YF-17D can predict the immunogenicity and T cell responses in PBMCs [37], but the early responses to YF-17D in DCs remain unveiled. To address this, we profiled the transcriptomes of the THP-1 cells, THP-1-derived iDCs, and THP-1-derived mDCs after YF-17D stimulation in vivo (Figure 2). A total number of 1570 differently expressed genes (DEGs) were identified, of which (a) 503 were upregulated and 151 downregulated in induction (Figure 2a); (b) 313 were upregulated and 864 downregulated in stimulation (Figure 2b); (c) 393 were changed only in induction, 916 only in stimulation, and 261 in both (Figure 2c). To investigate the mechanism of the DCs being stimulated by YF-17D, we analyzed 313 genes that were significantly upregulated during stimulation by enriching them in Kyoto Encyclopedia of Genes and Genomes (KEGG) [38,39], Reactome [40] and the Molecular Signatures Database Hallmark Gene Sets (MSigDB_H) [41]. As illustrated in Figure 2d, the transcriptional activities included pathogen-recognition receptor (PRR) activation (Toll-like receptors, NOD-like receptors, cytosolic DNA sensors), IFN-I-induced immunity (IFN signaling, IFN-stimulated genes, IFN-inducible proteins), cytokine signaling, etc.

We identified ten gene signatures representing the early immune responses stimulated by YF-17D in the DCs among those significantly enriched pathways (Figure 2e). Pattern recognition receptors upregulated IRF7 upon recognition of immunogenic YF-17D. Increased IRF7 expression activated the JAK/STAT signaling pathway [42], promoted IFN-I secretion, and further upregulated the expression levels of downstream IFN-inducible genes, including ISG15, OAS, MxA, IFIT, IFI, etc. The ubiquitin-like protein ISG15 directly inhibited virus replication [43] and modulated multiple signaling pathways in immunity [44]. The OAS2/3 proteins [45] generated 2′-5′-linked oligoadenylates to degrade exogenous RNA by activating the enzyme RNase L [45]. MxA inhibited virus transcription and replication by self-assembling into the viral ribonucleoprotein complex [46]. IFIT1 [47] and IFIT3 [48] acted as sensor and effector molecules targeting exogenous RNA. IFI27 stabilized the mitochondrial membrane potential [49]. IFI44 attached to nuclei to inhibit virus transcription [50]. SIGLEC1 was then activated via the JAK/STAT signaling pathway and suppressed IRF3 phosphorylation via TBK1 to inhibit IFN-I production [51]. In summary, the findings from the gene signatures and pathways point towards the dominant role of IFN-I-induced immunity in DCs stimulated by YF-17D. The activation of key proteins such as IRF7, ISG15, OAS2/3, MxA, IFIT1/3, IFI27/44, and SIGLEC1 suggests a robust early immune response to the YF-17D virus. These proteins play critical roles in inhibiting viral replication, sensing viral RNA, stabilizing the mitochondrial membrane potential, and regulating IFN-I production to prevent immunopathological disorders. These insights not only contribute to a better understanding of the early immune responses to YF-17D but also provide a predictive approach to evaluating vaccine immunogenicity.

### 2.3. Application of the TDDC Assessment Approach In Vitro and In Vivo

**Figure 3:**
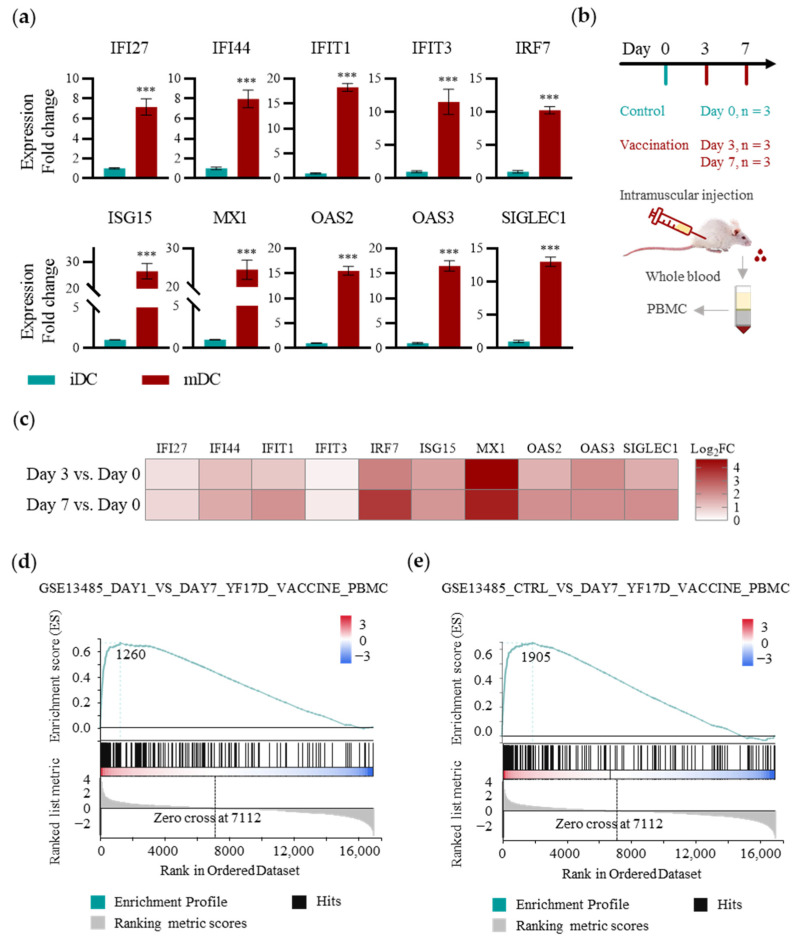
Validation of gene signatures in vivo and in vitro. (**a**) Relative expressions of gene signatures in THP-1-derived iDCs and YF-17D-stimulated mDCs via qPCR (*n* = 3). (**b**) Vaccination schedule of YF-17D in vivo for the gene signature profiling of early immune responses (*n* = 3). (**c**) Heatmap of gene signature expressions from essential pathways in PBMCs from YF-17D-vaccinated mice. Relative expressions were obtained via qPCR. (**d**,**e**) Gene Set Enrichment Analysis (GSEA) on MSigDB_C7 (immunologic signatures) with the transcriptomic variations in TDDCs after YF-17D stimulation. Statistical significance in (**a**) was determined using a two-tailed Student’s *t*-test. *** *p* < 0.001.

The use of in vitro TDDCs for immune response assessment can be beneficial for reducing genetic variation; however, these markers must also be detectable in PBMCs for further clinical application [31] (Figure 3). First, qPCR assays were used to determine the transcriptomic levels of these ten gene signatures before and after YF-17D stimulation of the TDDCs. The results showed that all these ten DEGs were significantly increased, which was consistent with the bulk RNA sequencing (RNA_seq), and that the increases were close to or greater than tenfold compared to their levels in the unstimulated TDDCs (Figure 3a).

Next, we extended the detection of gene signatures from the TDDCs to PBMC samples from model animals (Figure 3b). Mice were randomized into one control group and two experimental groups. The mice in the control group were not vaccinated, and whole blood was collected from their hearts on day 0; the mice in the experimental groups were vaccinated intramuscularly with YF-17D according to protocol, and one group was sampled on day 3 after vaccination and the other on day 7. PBMCs were isolated from the whole blood samples, and qPCR assays were used to detect gene expression (Figure 3c). The upregulation of MX1 was most significant on day 3 and day 7, indicating that activities to inhibit viral transcription were active. IRF7 was significantly upregulated on day 3 and continued to be elevated on day 7, indicating the positive regulation of IFN-I immunity. Most of the downstream gene signatures of IFN-I immunity, such as ISG15, OAS, and IFN-induced proteins, were already upregulated on day 3 and continued to be enhanced or maintained on day 7, indicating that IFN-I immune responses to YF-17D were progressively effective within a week. Moreover, the continued upregulation of SIGLEC1 demonstrated that the inhibitory mechanism against IFN-I immunity was gradually effective, implying that the activity of innate immunity tended to peak. Notably, the upregulation of the gene signatures detected in the mouse PBMC samples was significantly lower than that that in the TDDCs, demonstrating a reduction in the significance of the immune response signatures of the DCs due to their limited numerical percentage in the PBMCs. Except for IFIT3, nine of the ten gene signatures were successfully validated in the mouse PBMC samples, indicating that most of the gene signatures of the DCs were significant regardless of their low proportion in the PBMCs.

To validate the simulation of clinical situations using the TDDCs, we performed a Gene Set Enrichment Analysis (GSEA) [52] using MSigDB_C7 (immunologic signatures) with the transcriptome of the TDDCs. The enrichment results revealed that two of the strongest matches were comparisons with the GSE13485 dataset, which were upregulated DEGs in the PBMCs from day 7 after YF-17D vaccination versus the day 1 samples (ES = 0.667; Figure 3d) and the day 7 PBMCs versus the unvaccinated samples (ES = 0.646; Figure 3e), indicating consistency between the stimulated TDDCs and the PBMC samples from the clinical studies. In conclusion, we double-checked the gene signatures of the DCs in early-stage immunity and validated the potential of using the TDDC model to profile the early immune responses of YF-17D-vaccinated mice and clinical YF-17D vaccine recipients. 

### 2.4. Assessment of Early Immune Responses to Various Types of Vaccines of Human PBMCs

**Figure 4:**
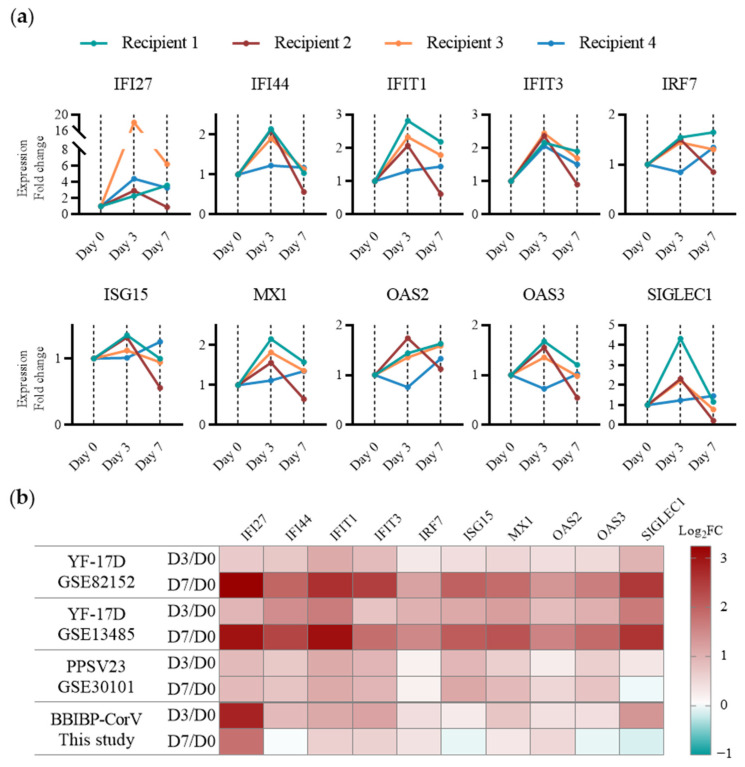
Assessment of early immune responses in dendritic cells (DCs) induced by vaccines. (**a**) Relative gene signature expressions in human PBMCs from BBIBP-CorV recipients via bulk RNA sequencing (*n* = 4). Each color stands for an individual recipient. (**b**) Heatmap of normalized gene signature expressions in human PBMCs from recipients vaccinated with various vaccines.

The YF-17D vaccine used to identify immunogenic biomarkers in this study is a live-attenuated vaccine. To study the general applicability of these biomarkers to different types of vaccines, we examined their serum levels in clinical samples vaccinated with an inactivated SARS-CoV-2 vaccine BBIBP-CorV, a live-attenuated yellow fever vaccine YF-17D, and a pneumococcal polysaccharide vaccine PPSV23 (Figure 4).

We firstly assessed the activation of human PBMCs by BBIBP-CorV according to our immunogenic biomarkers. Four volunteers were recruited (three male and one female), and whole blood samples were collected on day 0 before vaccination and day 3 and day 7 after vaccination. PBMCs were isolated, and RNA_seq was performed to determine their gene expression (Figure 4a). The overall trend showed that the IFN-I-induced immunity of recipients 1–3 was significantly enhanced on day 3 and decreased on day 7; on the other hand, the early immune responses of recipient 4 were consistently insignificant, showing the heterogeneity of the immune system in response to vaccine stimulation. Specifically, with reference to IRF7 and SIGLEC1, two IFN-I immunity regulatory genes, the IFN-I-induced immune response was most active in recipient 1 and slightly lower in recipients 2 and 3, which was also consistent with the expression of the other IFN-induced proteins. Clinical studies usually examine neutralizing antibody levels dozens of days after vaccination to assess vaccine efficacy [9,11,53,54,55]. The early-stage immunity profile of DCs established in this study allows for the assessment of early immune response in vaccine recipients within 7 days, providing novel support for clinical research and vaccine development.

We further investigated published clinical data from clinical samples stimulated by YF-17D [28,36], PPSV23 [56], and BBIBP-CorV vaccines. As shown in Figure 4b, the gene signatures of the early immune responses in the two YF-17D vaccine studies were consistent with the results obtained in the TDDCs and mouse PBMCs. This provides further evidence that the profiling of immune responses in DCs using the TDDC model is in accordance with the in vivo situation. The level of IFN-I immune response after PPSV23 vaccination was similar on day 3 and 7, where IRF7 was weaker than YF-17D and BBIBP-CorV; IFN-inducible proteins were significantly higher, to a similar degree to YF-17D on day 3; and OAS and ISG15 were slightly higher than with the other two vaccines. Unlike those in response YF-17D, which were continuously enhanced, and PPSV23, which were stable, the innate immune responses induced by BBIBP-CorV vaccination were stronger on day 3 than day 7. Notably, some gene signatures, such as ISG15, OAS3, and SIGLEC1, showed downregulation on day 7, implying limited activation of the immune responses by IFN-I. As a proof-of-concept analysis, we compared the transcriptome profiles of different clinical studies in the database to describe the early immune responses to vaccination. However, the populations, test protocols, and assay techniques varied from study to study. Critically, to assess the early immunogenicity profile of DCs against different vaccine candidates, variables should be controlled within the same framework, which can be achieved during vaccine development. 

Here, we identified a series of biomarkers of innate immunity in TDDCs and performed a proof-of-concept experiment with PBMC samples. However, systems biological assessment of immunity using PBMCs has several limitations [14]: (a) the changes observed in gene expression or signaling pathways may not be specific to a particular cell type due to cellular heterogeneity (for example, the IFN-I-induced immune responses identified in DCs may also be significant for other cells, such as monocytes and macrophages); (b) the immune response in the PBMCs may vary over time, and analysis at a single time point may not capture the dynamic changes; and (c) immune responses can vary significantly between individuals, and using PBMCs from different donors may introduce variability that complicates the data interpretation. Therefore, to comprehensively predict the performance of vaccines according to biomarkers, we need to further investigate other types of immune cells involved in innate immunity, set more sampling time points, increase the capacity of the clinical samples, compare the expression of biomarkers with the subsequent neutralizing antibody levels and other indicators, and ultimately establish rigorous algorithms to achieve the application of biomarkers for rapid and effective screening of vaccine candidates in vaccine development.

## 3. Materials and Methods

### 3.1. Materials

All the reagents were purchased from commercial sources and used without further purification. The THP-1 cell line was purchased from CAS Kunming Cell Bank, Kunming, China. The T-25 flasks and six-well plates were purchased from Corning, New York, NY, USA. RPMI 1640 and fetal bovine serum (FBS) were purchased from Gibco, New York, NY, USA. Recombinant human interleukin 4 (rhIL-4), recombinant human granulocyte–macrophage colony-stimulating factor (rhGM-CSF), recombinant human tumor necrosis factor α (rhTNF-α), and ionomycin were purchased from Beyotime, Shanghai, China. The YF-17D and BBIBP-CorV vaccines were obtained from Sinopharm, Beijing, China. TRIzol was purchased from Thermo Fisher, Waltham, MA, USA. The AG RNAex Pro Reagent, Evo M-MLV RT Kit, and SYBR Green Premix Pro Taq HS qPCR Kit were purchased from Accurate, Changsha, China. The human polyclonal anti-CD209 and human monoclonal anti-CD83 were purchased from Proteintech, Wuhan, China. Fluorescent polystyrene beads were purchased from NanoMicro, Suzhou, China. EDAC and NHS were purchased from Sigma-Aldrich, St. Louis, MO, USA. The flow cytometry reagents were purchased from BD Biosciences, San Jose, CA, USA. EDTAK2 vacuum blood collection tubes were purchased from Sanli, Liuyang, China. The medical-level density gradient reagent for PBMC isolation was purchased from Haoyang, Tianjin, China. Fluorescein isothiocyanate (FITC) was purchased from Yeasen, Shanghai, China. Milli-Q grade (>18 MΩ) water with sterilization was used throughout the experiments.

### 3.2. Cell Culture, Induction, and Stimulation

The THP-1 cells were cultured in RPMI 1640 medium supplemented with 10% FBS in 5% CO_2_ at 37 °C. For induction, the THP-1 cells were harvested and resuspended in an RPMI 1640 medium containing 10% FBS, 100 ng/mL of rhIL-4, and 100 ng/mL of rhGM-CSF in a six-well plate for 6 days, with the medium exchanged every two days. For stimulation with rhTNF-α, the THP-1-derived iDCs were harvested and resuspended in a serum-free RPMI 1640 medium containing 200 ng/mL of rhIL-4, 100 ng/mL of rhGM-CSF, 20 ng/mL of rhTNF-α, and 200 ng/mL of ionomycin for 24 h. For stimulation with the YF-17D vaccine, the YF-17D vaccine diluted with phosphate buffer saline (PBS) was added to the serum-free RPMI 1640 medium for the vaccination group, and an equal volume of PBS was added for the control group. The culture medium for YF-17D stimulation did not include rhIL-4, rhGM-CSF, rhTNF-α, or ionomycin.

### 3.3. RNA Preparation and qRT-PCR Testing

Cultured cells (>5 × 10^6^ cells) or PBMCs isolated from 500 μL of whole blood (>500 μL) were mixed with 1 mL of TRIzol immediately, and RNA was extracted according to the instructions for use and stored at −80 °C. The concentration and quality of the RNA were assessed using a NanoDrop 2000 spectrometer (Thermo Fisher, Waltham, MA, USA). Qualified RNA samples were processed with the Evo M-MLV RT Kit with gDNA Clean for qPCR into cDNA and stored at −20 °C. The SYBR Green Premix Pro Taq HS qPCR Kit was applied to perform the qPCR detection on a qTOWER3 system (Analytik Jena, Jena, Germany). The primers designed for the molecular markers are described in Tables S1–S3.

### 3.4. Flow Cytometry

Polystyrene microspheres consisting of FITC (488/520 nm) were used as the fluorescent labels. The buffer of the microsphere suspension was exchanged with MES, and EDC and NHS solution was added for activation. The antibody solution was mixed with the activated microsphere suspension solution at 4 °C for 20 h. The microspheres were blocked by PBS with 5% BSA and 1 mM glycine and stored at 4 °C after washing. The cells were fixed with 4% paraformaldehyde, washed with PBS, and mixed with antibody-coated fluorescent microsphere reagent at 4 °C for 30 min, avoiding light. The prepared cells were washed 2 times, resuspended at a density of 0.5–1 × 10^6^ cells/mL, and measured using a LSRFortessa flow cytometer (BD Biosciences, San Jose, CA, USA).

### 3.5. Transcriptomic Analysis

The THP-1 cells were seeded into 6-well plates, with each well containing over 2 million cells. Three duplicates of the THP-1 cells, THP-1-derived iDCs, and YF-17D-stimulated mDCs were collected. Total RNA was isolated, and bulk sequencing was performed. RNA sequencing was performed by BGI Genomics using a DNBSEQ sequencer (BGI). Differential expression analysis was performed on the count data using the R package DESeq2 1.21.9. The *p*-values obtained in the multiple binomial tests were adjusted using the FDR to achieve *q*-values. Differently expressed genes (DEGs) were defined according to a *q*-value of cut-off of 0.05 and |Log2FC| > 1 for the transcriptomic analysis. Multiple enrichments were performed according to a series of annotation systems, with a *q*-value ≤ 0.05 as the cut-off value for significance. GSEA was performed against the MSigDB_C7 (immunologic signatures) dataset using GSEA v4.1.0 software. The datasets presented in this study can be found in the online repositories the Sequence Read Archive (SRA, https://www.ncbi.nlm.nih.gov/sra/ accessed on date 15 May 2024) and BioProject (ID: PRJNA775870).

### 3.6. Animal Study

The animal studies were approved by the Ethics Committee of the Tsinghua Shenzhen International Graduate School ([2020 No.3]; 5 January 2020) and were performed according to the Guideline of Care and Use of Laboratory Animals. BALB/c mice (male, 4–6 weeks) were purchased from the Guangdong Medical Laboratory Animal Center and maintained in the animal center of Tsinghua Shenzhen International Graduate School. Nine BALB/c mice aged 6–8 weeks were assigned into 3 groups equally, labeled A, B, and C. The mice in group A were injected intramuscularly (quadriceps) with 60 μL of saline, while the mice in groups B and C were injected intramuscularly (quadriceps) with 60 μL of the diluted YF-17D vaccine, containing 1% of the immunogenic components of the standard dose. Over 500 μL of whole blood from each mouse was collected from the heart on day 0 for group A, day 3 for group B, and day 7 for group C. The PBMCs were isolated with medical-level density gradient reagents, mixed with 1 mL of TRIzol immediately, and stored at −80 °C. 

### 3.7. Clinical Study

The Ethics Committee of the Tsinghua Shenzhen International Graduate School ([2021 No.35]; 31 March 2021) approved this study for immunogenicity estimation of the inactivated SARS-CoV-2 vaccine BBIBP-CorV. Four volunteers aged 20–40 were recruited from students on Shenzhen University Town campus. The volunteers were enrolled in the BBIBP-CorV vaccination campaign operated by the government and received 2 injections in March and April 2021, respectively. Their whole blood (2–3 mL) was collected in EDTAK2 vacuum blood collection tubes on the day before vaccination (day 0) and on days 3 and 7 after vaccination. Their PBMCs were isolated with medical-level density gradient reagent, mixed with 1 mL of AG RNAex Pro Reagent, and stored at −80 °C. 

### 3.8. Statistics

All data are presented as means ± SD. Statistical significance was calculated via two-way analysis of variance (ANOVA) with Dunnett’s test and a two-tailed Student’s *t*-test using GraphPad Prism 9.3.1 software. For the statistical analysis, * *p* < 0.05, ** *p* < 0.01, and *** *p* < 0.001.

## 4. Conclusions

In conclusion, the findings of this study demonstrate the potential of using a TDDC model to profile early immune responses and identify biomarkers. The assessment of early immune responses to various types of vaccines also revealed the dynamic nature of IFN-I-induced immunity and the heterogeneity of immune system responses to different vaccine stimulations. Compared to traditional neutralizing antibody assays, these results highlight the importance of early-stage immunity profiling in understanding the effectiveness of vaccines in the short term. The consistent gene signatures of early immune responses in the TDDCs, mouse PBMCs, and clinical samples stimulated by the YF-17D, PPSV23, and BBIBP-CorV vaccines further support the relevance and applicability of the biomarkers identified. These findings also emphasize the need for standardized frameworks in assessing the early immunogenicity profiles of DCs in response to different vaccine candidates, particularly during vaccine development processes. Overall, the comprehensive analysis presented in this study contributes to advancing our understanding of early immune responses to vaccination and underscores the potential of utilizing a TDDC model to identify immunogenic biomarkers across different vaccine types. This insight holds the promise of informing and accelerating the development of effective vaccines for various infectious diseases.

## Data Availability

Data are available upon request.

## Supplementary Materials

The following supporting information can be downloaded at https://www.mdpi.com/article/10.3390/ijms25105509/s1.

## References

[1] Mishra S.K., Tripathi T. (2021). One Year Update on the COVID-19 Pandemic: Where Are We Now?. Acta Trop..

[2] Long Q.-X., Liu B.-Z., Deng H.-J., Wu G.-C., Deng K., Chen Y.-K., Liao P., Qiu J.-F., Lin Y., Cai X.-F. (2020). Antibody Responses to SARS-CoV-2 in Patients with COVID-19. Nat. Med..

[3] Sahin U., Muik A., Derhovanessian E., Vogler I., Kranz L.M., Vormehr M., Baum A., Pascal K., Quandt J., Maurus D. (2020). COVID-19 Vaccine BNT162b1 Elicits Human Antibody and TH1 T Cell Responses. Nature.

[4] Mulligan M.J., Lyke K.E., Kitchin N., Absalon J., Gurtman A., Lockhart S., Neuzil K., Raabe V., Bailey R., Swanson K.A. (2020). Phase I/II Study of COVID-19 RNA Vaccine BNT162b1 in Adults. Nature.

[5] Abu-Raddad L.J., Chemaitelly H., Butt A.A. (2021). Effectiveness of the BNT162b2 Covid-19 Vaccine against the B.1.1.7 and B.1.351 Variants. N. Engl. J. Med..

[6] Shahapur P.R., Shahapur R., Bagali S., Karigoudar R., Wavare D.S., P J., Kandi V., Suvvari T.K., Mittal R.J., Jadhav M. (2022). Breakthrough Infections: Clinical Profile and Outcomes of COVID-19 Vaccinated and Unvaccinated People from a Tertiary Care Hospital. Cureus.

[7] Ma H., Zhao D., Zeng W., Yang Y., Hu X., Zhou P., Weng J., Cheng L., Zheng X., Jin T. (2021). Decline of SARS-CoV-2-Specific IgG, IgM and IgA in Convalescent COVID-19 Patients within 100 Days after Hospital Discharge. Sci. China Life Sci..

[8] Criscuolo E., Diotti R.A., Strollo M., Rolla S., Ambrosi A., Locatelli M., Burioni R., Mancini N., Clementi M., Clementi N. (2021). Weak Correlation between Antibody Titers and Neutralizing Activity in Sera from SARS-CoV-2 Infected Subjects. J. Med. Virol..

[9] Khoury D.S., Cromer D., Reynaldi A., Schlub T.E., Wheatley A.K., Juno J.A., Subbarao K., Kent S.J., Triccas J.A., Davenport M.P. (2021). Neutralizing Antibody Levels Are Highly Predictive of Immune Protection from Symptomatic SARS-CoV-2 Infection. Nat. Med..

[10] Stravalaci M., Pagani I., Paraboschi E.M., Pedotti M., Doni A., Scavello F., Mapelli S.N., Sironi M., Perucchini C., Varani L. (2022). Recognition and Inhibition of SARS-CoV-2 by Humoral Innate Immunity Pattern Recognition Molecules. Nat. Immunol..

[11] Corti D., Purcell L.A., Snell G., Veesler D. (2021). Tackling COVID-19 with Neutralizing Monoclonal Antibodies. Cell.

[12] Pulendran B. (2014). Systems Vaccinology: Probing Humanity’s Diverse Immune Systems with Vaccines. Proc. Natl. Acad. Sci. USA.

[13] Hagan T., Nakaya H.I., Subramaniam S., Pulendran B. (2015). Systems Vaccinology: Enabling Rational Vaccine Design with Systems Biological Approaches. Vaccine.

[14] Arunachalam P.S., Wimmers F., Mok C.K.P., Perera R., Scott M., Hagan T., Sigal N., Feng Y., Bristow L., Tak-Yin Tsang O. (2020). Systems Biological Assessment of Immunity to Mild versus Severe COVID-19 Infection in Humans. Science.

[15] Upreti S., Samant M. (2022). A Review on Immunological Responses to SARS-CoV-2 and Various COVID-19 Vaccine Regimens. Pharm. Res..

[16] Maecker H.T., McCoy J.P., Nussenblatt R. (2012). Standardizing Immunophenotyping for the Human Immunology Project. Nat. Rev. Immunol..

[17] Steinman R.M. (1991). The Dendritic Cell System and Its Role in Immunogenicity. Annu. Rev. Immunol..

[18] Banchereau J., Briere F., Caux C., Davoust J., Lebecque S., Liu Y.-J., Pulendran B., Palucka K. (2000). Immunobiology of Dendritic Cells. Annu. Rev. Immunol..

[19] Collin M., Bigley V. (2018). Human Dendritic Cell Subsets: An Update. Immunology.

[20] Sallusto F., Lanzavecchia A. (1994). Efficient Presentation of Soluble Antigen by Cultured Human Dendritic Cells Is Maintained by Granulocyte/Macrophage Colony-Stimulating Factor plus Interleukin 4 and Downregulated by Tumor Necrosis Factor Alpha. J. Exp. Med..

[21] Romani N., Gruner S., Brang D., Kämpgen E., Lenz A., Trockenbacher B., Konwalinka G., Fritsch P.O., Steinman R.M., Schuler G. (1994). Proliferating Dendritic Cell Progenitors in Human Blood. J. Exp. Med..

[22] Cheong C., Matos I., Choi J.H., Dandamudi D.B., Shrestha E., Longhi M.P., Jeffrey K.L., Anthony R.M., Kluger C., Nchinda G. (2010). Microbial Stimulation Fully Differentiates Monocytes to DC-SIGN/CD209(+) Dendritic Cells for Immune T Cell Areas. Cell.

[23] Johnson J.S., De Veaux N., Rives A.W., Lahaye X., Lucas S.Y., Perot B.P., Luka M., Garcia-Paredes V., Amon L.M., Watters A. (2020). A Comprehensive Map of the Monocyte-Derived Dendritic Cell Transcriptional Network Engaged upon Innate Sensing of HIV. Cell Rep..

[24] Rigamonti A., Villar J., Segura E. (2023). Monocyte Differentiation within Tissues: A Renewed Outlook. Trends Immunol..

[25] Akira S., Uematsu S., Takeuchi O. (2006). Pathogen Recognition and Innate Immunity. Cell.

[26] Flynn J.L., Chan J. (2022). Immune Cell Interactions in Tuberculosis. Cell.

[27] Gordon S., Taylor P.R. (2005). Monocyte and Macrophage Heterogeneity. Nat. Rev. Immunol..

[28] Querec T.D., Akondy R.S., Lee E.K., Cao W., Nakaya H.I., Teuwen D., Pirani A., Gernert K., Deng J., Marzolf B. (2009). Systems Biology Approach Predicts Immunogenicity of the Yellow Fever Vaccine in Humans. Nat. Immunol..

[29] Berges C., Naujokat C., Tinapp S., Wieczorek H., Höh A., Sadeghi M., Opelz G., Daniel V. (2005). A Cell Line Model for the Differentiation of Human Dendritic Cells. Biochem. Biophys. Res. Commun..

[30] Hitzler M., Bergert A., Luch A., Peiser M. (2013). Evaluation of Selected Biomarkers for the Detection of Chemical Sensitization in Human Skin: A Comparative Study Applying THP-1, MUTZ-3 and Primary Dendritic Cells in Culture. Toxicol. Vitr..

[31] Chanput W., Mes J.J., Wichers H.J. (2014). THP-1 Cell Line: An in Vitro Cell Model for Immune Modulation Approach. Int. Immunopharmacol..

[32] Li L., Wang S., Zou Z., Tao A., Ai Y. (2018). Activation Profile of THP-1 Derived Dendritic Cells Stimulated by Allergen Mal f 1 beyond Its IgE-Binding Ability. Int. Immunopharmacol..

[33] Lechmann M., Berchtold S., Steinkasserer A., Hauber J. (2002). CD83 on Dendritic Cells: More than Just a Marker for Maturation. Trends Immunol..

[34] Zhou L.J., Tedder T.F. (1996). CD14+ Blood Monocytes Can Differentiate into Functionally Mature CD83+ Dendritic Cells. Proc. Natl. Acad. Sci. USA.

[35] Barrett A.D.T., Monath T.P., Barban V., Niedrig M., Teuwen D.E. (2007). 17D Yellow Fever Vaccines: New Insights. Vaccine.

[36] Hou J., Wang S., Jia M., Li D., Liu Y., Li Z., Zhu H., Xu H., Sun M., Lu L. (2017). A Systems Vaccinology Approach Reveals Temporal Transcriptomic Changes of Immune Responses to the Yellow Fever 17D Vaccine. J. Immunol..

[37] Pulendran B. (2009). Learning Immunology from the Yellow Fever Vaccine: Innate Immunity to Systems Vaccinology. Nat. Rev. Immunol..

[38] Kanehisa M. (2000). KEGG: Kyoto Encyclopedia of Genes and Genomes. Nucleic Acids Res..

[39] Kanehisa M., Furumichi M., Sato Y., Kawashima M., Ishiguro-Watanabe M. (2023). KEGG for Taxonomy-Based Analysis of Pathways and Genomes. Nucleic Acids Res..

[40] Gillespie M., Jassal B., Stephan R., Milacic M., Rothfels K., Senff-Ribeiro A., Griss J., Sevilla C., Matthews L., Gong C. (2022). The Reactome Pathway Knowledgebase 2022. Nucleic Acids Res..

[41] Liberzon A., Birger C., Thorvaldsdóttir H., Ghandi M., Mesirov J.P., Tamayo P. (2015). The Molecular Signatures Database Hallmark Gene Set Collection. Cell Syst..

[42] Honda K., Taniguchi T. (2006). IRFs: Master Regulators of Signalling by Toll-like Receptors and Cytosolic Pattern-Recognition Receptors. Nat. Rev. Immunol..

[43] Perng Y.-C., Lenschow D.J. (2018). ISG15 in Antiviral Immunity and Beyond. Nat. Rev. Microbiol..

[44] Kim D.H., Ewbank J.J. (2018). Signaling in the Innate Immune Response. WormBook.

[45] Hornung V., Hartmann R., Ablasser A., Hopfner K.-P. (2014). OAS Proteins and cGAS: Unifying Concepts in Sensing and Responding to Cytosolic Nucleic Acids. Nat. Rev. Immunol..

[46] Fensterl V., Wetzel J.L., Ramachandran S., Ogino T., Stohlman S.A., Bergmann C.C., Diamond M.S., Virgin H.W., Sen G.C. (2012). Interferon-Induced Ifit2/ISG54 Protects Mice from Lethal VSV Neuropathogenesis. PLoS Pathog..

[47] Diamond M.S. (2014). IFIT1: A Dual Sensor and Effector Molecule That Detects Non-2′-O Methylated Viral RNA and Inhibits Its Translation. Cytokine Growth Factor. Rev..

[48] Liu X.-Y., Chen W., Wei B., Shan Y.-F., Wang C. (2011). IFN-Induced TPR Protein IFIT3 Potentiates Antiviral Signaling by Bridging MAVS and TBK1. J. Immunol..

[49] Cheriyath V., Leaman D.W., Borden E.C. (2011). Emerging Roles of FAM14 Family Members (G1P3/ISG 6–16 and ISG12/IFI27) in Innate Immunity and Cancer. J. Interferon Cytokine Res..

[50] Power D., Santoso N., Dieringer M., Yu J., Huang H., Simpson S., Seth I., Miao H., Zhu J. (2015). IFI44 Suppresses HIV-1 LTR Promoter Activity and Facilitates Its Latency. Virology.

[51] Zheng Q., Hou J., Zhou Y., Yang Y., Xie B., Cao X. (2015). Siglec1 Suppresses Antiviral Innate Immune Response by Inducing TBK1 Degradation via the Ubiquitin Ligase TRIM27. Cell Res..

[52] Subramanian A., Tamayo P., Mootha V.K., Mukherjee S., Ebert B.L., Gillette M.A., Paulovich A., Pomeroy S.L., Golub T.R., Lander E.S. (2005). Gene Set Enrichment Analysis: A Knowledge-Based Approach for Interpreting Genome-Wide Expression Profiles. Proc. Natl. Acad. Sci. USA.

[53] Liu Y., Liu J., Xia H., Zhang X., Fontes-Garfias C.R., Swanson K.A., Cai H., Sarkar R., Chen W., Cutler M. (2021). Neutralizing Activity of BNT162b2-Elicited Serum. N. Engl. J. Med..

[54] Planas D., Veyer D., Baidaliuk A., Staropoli I., Guivel-Benhassine F., Rajah M.M., Planchais C., Porrot F., Robillard N., Puech J. (2021). Reduced Sensitivity of SARS-CoV-2 Variant Delta to Antibody Neutralization. Nature.

[55] Wu K., Werner A.P., Koch M., Choi A., Narayanan E., Stewart-Jones G.B.E., Colpitts T., Bennett H., Boyoglu-Barnum S., Shi W. (2021). Serum Neutralizing Activity Elicited by mRNA-1273 Vaccine. N. Engl. J. Med..

[56] Obermoser G., Presnell S., Domico K., Xu H., Wang Y., Anguiano E., Thompson-Snipes L., Ranganathan R., Zeitner B., Bjork A. (2013). Systems Scale Interactive Exploration Reveals Quantitative and Qualitative Differences in Response to Influenza and Pneumococcal Vaccines. Immunity.

