# Supplementary material for: Profiling of Early Immune Responses to Vaccination Using THP-1-Derived Dendritic Cells
Source: Int J Mol Sci. 2024 May 18;25(10):5509. doi: 10.3390/ijms25105509 (PMC11121899; doi:10.3390/ijms25105509)
Supplement: Supplementary file 1 [file ijms-25-05509-s001.zip › ijms-2970443-supplementary.pdf]

Supporting Information

Profiling of Early Immune Responses to Vaccination Using THP-1-Derived Dendritic Cells

Lei Ye [1,2,†], Ping Li [1,†], Mingzhe Wang [1], Feng Wu [1], Sanyang Han [1,*] and Lan Ma [1,2,3,*]

[1] Institute of Biopharmaceutical and Health Engineering, Tsinghua Shenzhen International Graduate School, Tsinghua University, Shenzhen 518055, China; yelei@szbl.ac.cn (L.Y.); lipinga104@163.com (P.L.); wang-mz17@mails.tsinghua.edu.cn (M.W.); wf19@mails.tsinghua.edu.cn (F.W.)

[2] Institute of Biomedical Health Technology and Engineering, Shenzhen Bay Laboratory, Shenzhen 518052, China

[3] State Key Laboratory of Chemical Oncogenomics, Tsinghua Shenzhen International Graduate School, Tsinghua University, Shenzhen 518055, China

* Correspondence: hansanyang@sz.tsinghua.edu.cn (S.H.); malan@sz.tsinghua.edu.cn (L.M.)

† These authors contributed equally to this article.

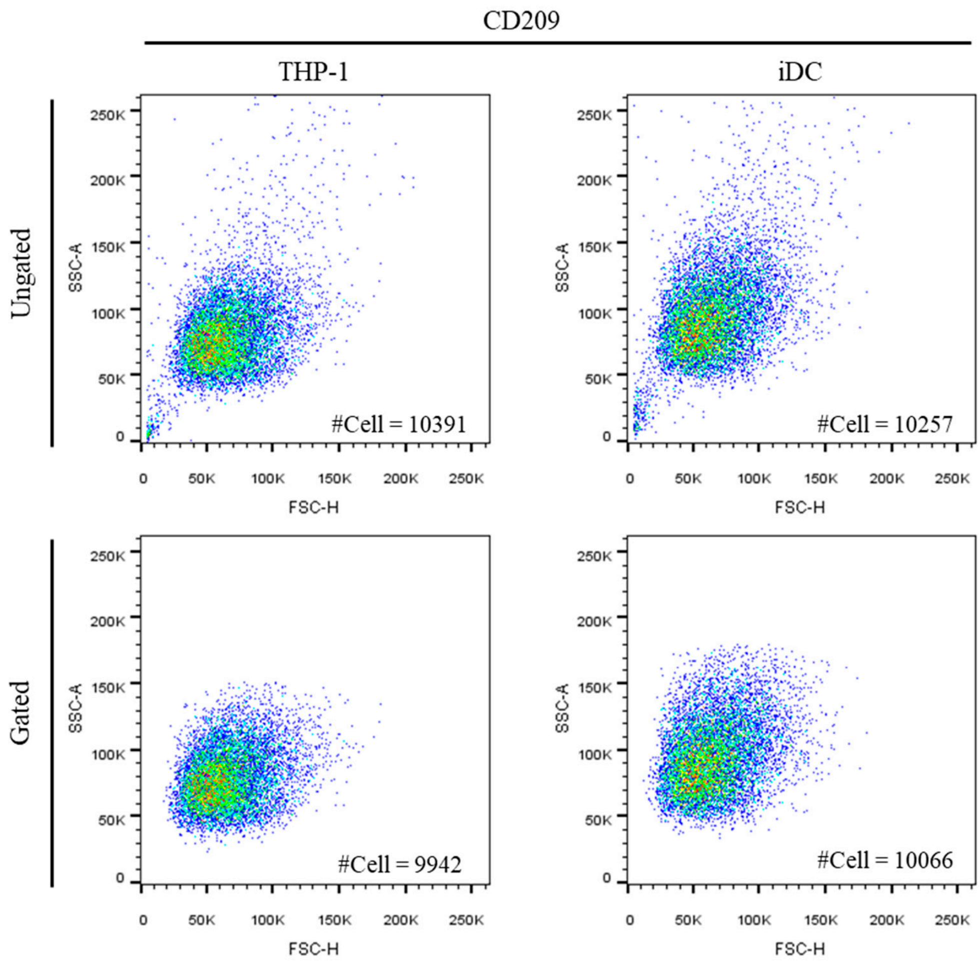

Figure S1. Flow cytometry gating for THP-1 and THP-1-derived iDC cells single stained for CD209.

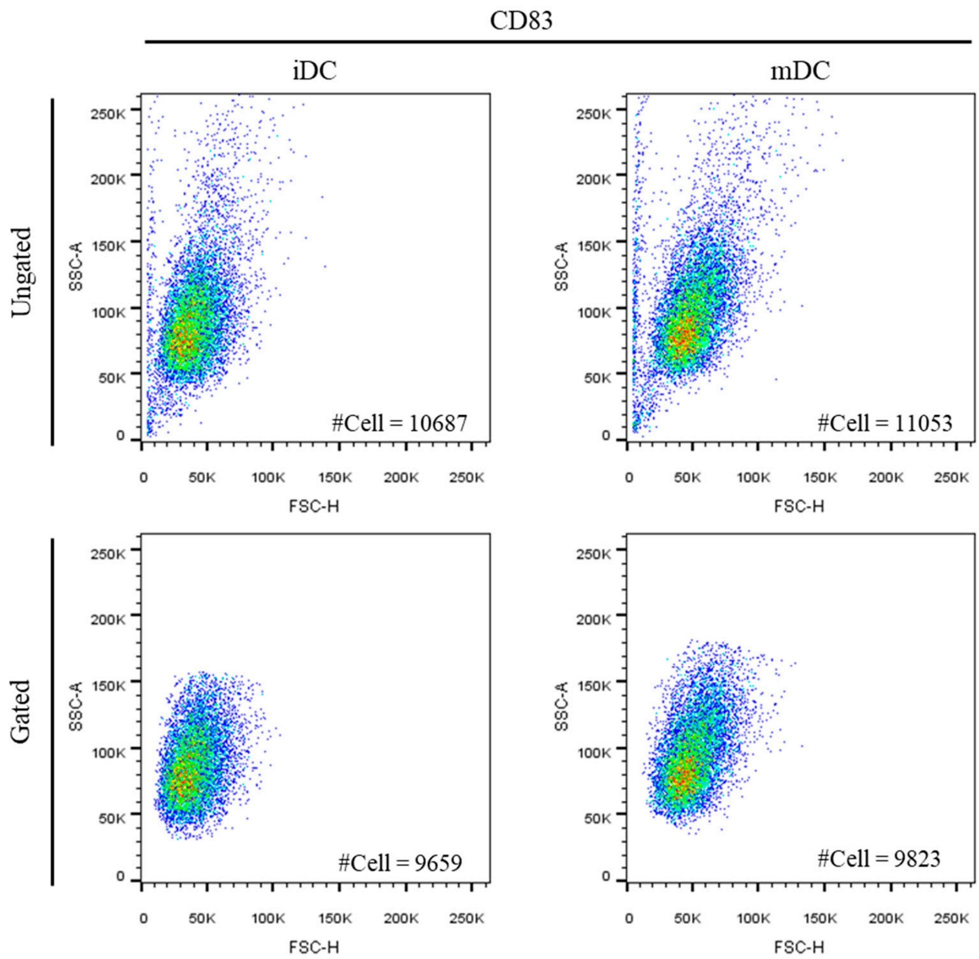

Figure S2. Flow cytometry gating for THP-1-derived iDC and mDC cells single stained for CD83.

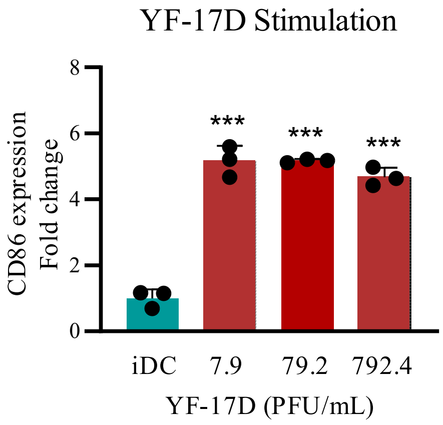

Figure S3. Relative expression of CD86 mRNA in THP-1-derived iDCs and dendritic cells stimulated by 7.9, 79.2, 792.4 PFU/mL of YF-17D via qRT-PCR (n = 3). Data are represented as mean ± SD. Statistical significance was calculated via one-way analysis of variance (ANOVA) with a Dunnett's test. *p < 0.05, **p < 0.01, and ***p < 0.001.

Table S1. Primers for validating TDDCs model establishment

Biomarkers	Forward Primer (5′ to 3′)	Reverse Primer (5′ to 3′)
CD209	AATGGCTGGAACGACGACAAA	CAGGAGGCTGCGGACTTTTT
CD83	AAGGGGCAAAATGGTTCTTTCG	GCACCTGTATGTCCCCGAG
CD86	CTGCTCATCTATACACGGTTACC	GGAAACGTCGTACAGTTCTGTG
GAPDH	CTGGGCTACACTGAGCACC	AAGTGGTCGTTGAGGGCAATG

Table S2. Primers for gene signatures in TDDCs validation

Biomarkers	Forward Primer (5′ to 3′)	Reverse Primer (5′ to 3′)
IFI27	TGCTCTCACCTCATCAGCAGT	CACAACTCCTCCAATCACAACT
IFI44	ATGGCAGTGACAACTCGTTTG	TCCTGGTAACTCTCTTCTGCATA
IFIT1	GCGCTGGGTATGCGATCTC	CAGCCTGCCTTAGGGGAAG
IFIT3	TCAGAAGTCTAGTCACTTGGGG	ACACCTTCGCCCTTTCATTTC
IRF7	CCCACGCTATACCATCTACCT	GATGTCGTCATAGAGGCTGTTG
ISG15	TGGACAAATGCGACGAACCTC	TCAGCCGTACCTCGTAGGTG
MX1	AGCGGGATCGTGACCAGAT	TGACCTTGCCTCTCCACTTATC
OAS2	AGGTGGCTCCTATGGACGG	TTTATCGAGGATGTCACGTTGG
OAS3	GCTTCAAGAGCTATGTGGACC	GGAAACGTGAGTCTCAGACCA
SIGLEC1	CCTCGGGGAGGAACATCCTT	AGGCGTACCCCATCCTTGA
GAPDH	CTGGGCTACACTGAGCACC	AAGTGGTCGTTGAGGGCAATG

Table S3. Primers for gene signatures in mice PBMC detection

Biomarkers	Forward Primer (5′ to 3′)	Reverse Primer (5′ to 3′)
IFI27	GACTCTCCGTGCCATCTACTG	CCTCTATCGCCATATCTGCCAC
IFI44	AACTGACTGCTCGCAATAATGT	GTAACACAGCAATGCCTCTTGT
IFT1	CTGAGATGTCACTTCACATGGAA	GTGCATCCCCAATGGGTTCT
IFT3	CCTACATAAAGCACCTAGATGGC	ATGTGATAGTAGATCCAGGCGT
IRF7	GAGACTGGCTATTGGGGGAG	GACCGAAATGCTTCCAGGG
ISG15	GGTGTCCGTGACTAACTCCAT	TGGAAAGGGTAAGACCGTCCT
MX1	GACCATAGGGGTCTTGACCAA	AGACTTGCTCTTTCTGAAAAGCC
OAS2	TTGAAGAGGAATACATGCGGAAG	GGGTCTGCATTACTGGCACTT

OAS3	TCTGGGGTCGCTAAACATCAC	GATGACGAGTTCGACATCGGT
SIGLEC1	CAGGGCATCCTCGACTGTC	GGAGCATCGTGAAGTTGGTTG
GAPDH	AGGTCGGTGTGAACGGATTTG	TGTAGACCATGTAGTTGAGGTCA
